# A High Gain Circularly Polarized Slot Antenna Array for 5G Millimeter-Wave Applications

**DOI:** 10.3390/s24196175

**Published:** 2024-09-24

**Authors:** Wei He, Jun Hong, Yongmei Ren, Yuanxiang Deng, Xiaohu Wang, Xiaoyong Fang

**Affiliations:** 1 Institute of Human Factors and Safety Engineering, Hunan Institute of Technology, Hengyang 421002, China; hewei21801@163.com (W.H.); 13875741015@163.com (J.H.); 2 School of Electrical Information Engineering, Hunan Institute of Technology, Hengyang 421002, China; 2013001813@hnit.edu.cn; 3 School of Science, Hunan Institute of Technology, Hengyang 421002, China; yxdeng@hnu.edu.cn; 4 School of Intelligent Manufacturing and Mechanical Engineering, Hunan Institute of Technology, Hengyang 421002, China; 2003000285@hnit.edu.cn

**Keywords:** millimeter-wave antenna, circularly polarized antenna, substrate integrated waveguide, all-metal

## Abstract

An air-filled substrate-integrated waveguide (AF-SIW) circularly polarized (CP) 1 × 8 mm wave antenna array is presented for fifth-generation (5G) applications. The presented slot antenna array consists of three layers of PCB and one layer of aluminum, which serve as the AF-SIW feeding network and the metal cavity radiation element, respectively. The CP characteristic is achieved by the use of an *S*-shaped aluminum radiation cavity on the top of the AF-SIW feeding network. The air-filled substrate-integrated waveguide technique is unitized to achieve high radiation efficiency. A wide input impedance bandwidth of 18.4% is obtained for the proposed antenna scheme, ranging from 34.5 GHz to 41.5 GHz, with a peak gain of 18 dBic. As for CP characteristic, the proposed antenna possesses a wide 3 dB axial ratio (AR) bandwidth, which is 16.4% (36.5 GHz to 43 GHz). The antenna scheme is fabricated and measured to verify the potential application as well as the promising performance. The measured results of the 1 × 8 antenna array shows that the wide AR as well as the input impedance are simultaneously achieved, which coincide well with the simulated results. Also, the measured results indicate that the proposed antenna scheme might be a good candidate for future mobile applications.

## 1. Introduction

In recent years, with the continuous development of wireless communication systems, especially the large-scale commercial use of the fifth-generation mobile communication technology, millimeter-wave (MMW) technology has attracted more attention from academia and industry [1,2,3]. There have been increasing demands for MMW antennas/arrays in wireless communication applications due to their low cost, wide bandwidth, and high gain characteristics [4,5,6,7,8,9]. In another aspect, the circularly polarized (CP) antennas, especially for millimeter-wave applications, have also obtained considerable attention considering that circularly polarized antennas benefit from certain advantages over linearly polarized counterparts such as the suppression of a multipath effect and high receiving efficiency for terminal and anti-spin effects [10,11,12]. Hence, CP antennas have potential scenarios in various application fields, including satellite communication [13], wireless communication [14], navigation system [15], biomedical telemetry [16], and Internet of Things (IoT) technology [17].

Also, millimeter-wave systems prefer to use high-gain antenna arrays to counteract the severe path loss caused by refracted/reflected/transmitted processes. For MMW applications, typical feeding networks such as a microstrip-line feeding network may cause much dielectric loss and decrease the gain performance of the antenna arrays. Hence, the technology known as substrate-integrated waveguide (SIW) is widely regarded as a promising solution for easy integration, easy processing, high power capability, and low loss compared with microstrip-line feeding network [18,19,20]. The presence of a dielectric material in the propagation medium results in undesirable high dielectric losses, which would dramatically decrease the task performance of the MMW terminal, especially the radiation efficiency. Hence, removing the dielectric material from the feeding network and the radiation element might be a suitable method to decrease the dielectric loss and improve the radiation efficiency of the antenna/array. One solution to reduce the dielectric loss of SIW configuration is to replace other the dielectric fill with air fill, which is called an air-filled substrate-integrated waveguide (AF-SIW).

In this paper, a novel high-gain, AF-SIW CP slot antenna array is designed within 37–40 GHz for 5G mobile communication applications. Firstly, an AF-SIW CP slot antenna element is proposed with a metal cavity as a radiation structure. The metal cavity has two arms, which can produce circularly polarized radiation. Then, the AF-SIW is utilized as the feeding network to implement a 1 × 8 element CP slot antenna array. The results indicate that the proposed slot array exhibits a broad axial ratio (AR) bandwidth exceeding 13% (36–41 GHz). The peak gain of 18 dBic at 36.5 GHz is also obtained for the proposed AF-SIW CP slot antenna array. Finally, the left-hand CP and right-hand CP radiation patterns of the antenna scheme are predominantly symmetrical, achieving high gain boresight radiation characteristics. The design and development of the proposed antenna were achieved using the commercial electromagnetic (EM) software HFSS 18.1. The details of the antenna element and 1 × 8 antenna array design are introduced in Section 2 and Section 3, respectively, and Section 4 is the conclusion.

## 2. AF-SIW CP Slot Antenna Element

**Figure 1:**
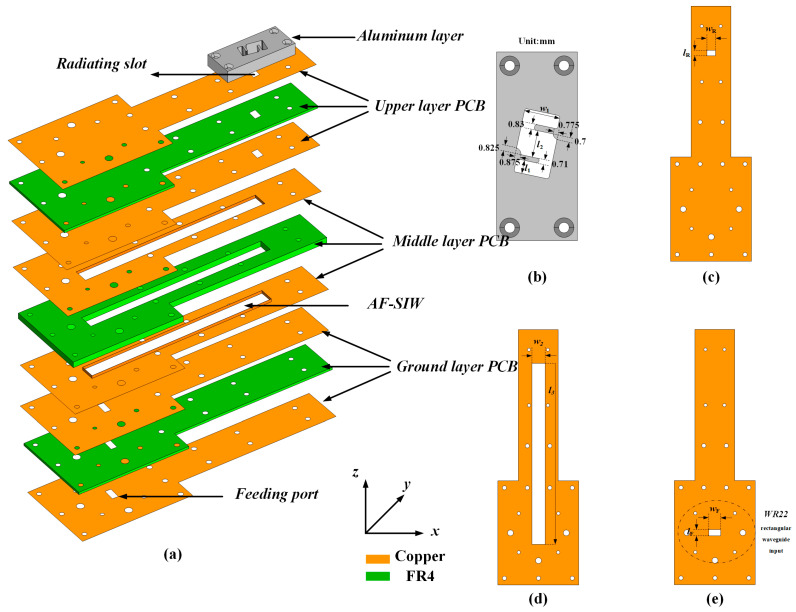
Geometry of the proposed antenna element: (**a**) 3D exploded view; (**b**) Top view of the aluminum layer; (**c**) Top view of the upper layer, (**d**) middle layer; (**e**) Top view of the ground layer.

Figure 1 depicts an exploded view of the proposed AF-SIW CP slot antenna element. The proposed antenna configuration is composed of three layers of PCB and an aluminum layer. The aluminum acts as the radiation structure of the antenna. The three layers of PCB are properly designed for better power transmission, acting as the waveguide feeding network by the SIW technique. As shown in Figure 1, the substrate of the three layers of PCB is made by the FR4 dielectric material, with a relative permittivity of 4.4 and a low loss tangent of 0.02, and the upper and lower surfaces of each layer of PCB are etched with copper. The overall thickness (including copper) of the upper PCB layer, the middle PCB layer, the ground PCB layer, and the aluminum layer are 0.4 mm, 2.4 mm, 0.4 mm, and 4.8 mm, respectively. The three PCB layers should be designed properly to achieve the desired AF-SIW feeding network configuration. Firstly, copper is etched on the bottom of the upper PCB layer, acting as the top waveguide wall of the AF-SIW configuration. Secondly, an empty cavity is hollowed out within the middle PCB layer to decrease the dielectric loss within the structure. Note that the four inner sidewalls of the empty structure are covered with copper (fabricated by the electroplating process), which can be deemed as the side waveguide walls of the AF-SIW. Finally, both the top and bottom faces of the ground PCB layer are etched with copper, and these two copper layers act as the bottom waveguide wall of the AF-SIW.

The detailed parameters of the different substrate and copper layers are depicted in Figure 1b–d. Figure 1b depicts the detailed configuration of the aluminum cavity. The 5 mm thick cavity is designed as S-shaped, where the width of the S-shaped slot is *w*_1_ and the length is *l*_2_. The S-shaped slot is responsible for the CP mechanism, which will be discussed in the next part. To couple energy from the AF-SIW feeding network configuration into the S-shaped cavity, a radiation slot is cut out on the upper substrate layer, and four sidewalls of the slot are covered with copper. The length and the width of the radiation slot are *w*_R_ and *l*_R_, respectively, which could be observed in Figure 1c. Also, the width of the AF waveguide is *w*_2_, which is illustrated in Figure 1d. Then, another slot is etched into the bottom of the ground layer. The parameters and the fork of the slot coincide well with the standard WR22 rectangular waveguide input configuration shown in Figure 1e. Finally, the detailed parameters of the abovementioned structures are shown in Table 1.

**Figure 2:**
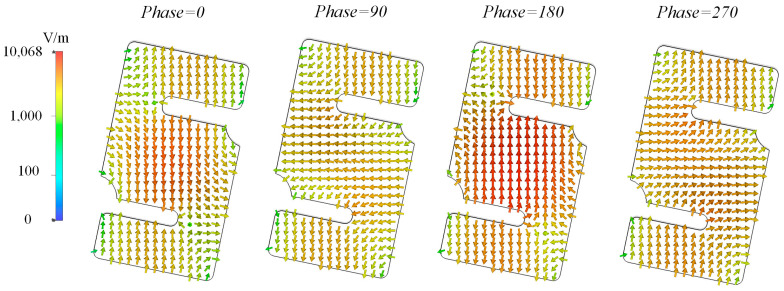
Simulated results of time-varying E-fields inside S-shaped cavity of the proposed CP antenna element at 38 GHz.

In order to better understand the circularly polarized mechanism of the proposed CP slot antenna element, the simulated E-field distributions within the aluminum cavity at 38 GHz with different phases are displayed in Figure 2. Due to the angle between the S-shaped cavity and the radiation slot on the upper substrate layer, the E-field distribution is disturbed by the aluminum. Hence, the radiation E-field source would rotate with the phase variation, indicating that the orthogonal radiation source with an equal amplitude would be achieved if the structure as well as the arrangement of the aluminum cavity is properly designed. As depicted in Figure 2, the E-field distribution is along the -*y* direction at phase = 0°, along the -*x* direction at phase = 90°, along the +y direction at phase = 180°, and along the +x direction at phase = 270°. The E-field distribution rotates in the clockwise direction, which indicates that a left-hand circular polarization (LHCP) wave is generated along the +z direction. Consequently, the LHCP radiation characteristics could be achieved by the proposed antenna element.

**Figure 3:**
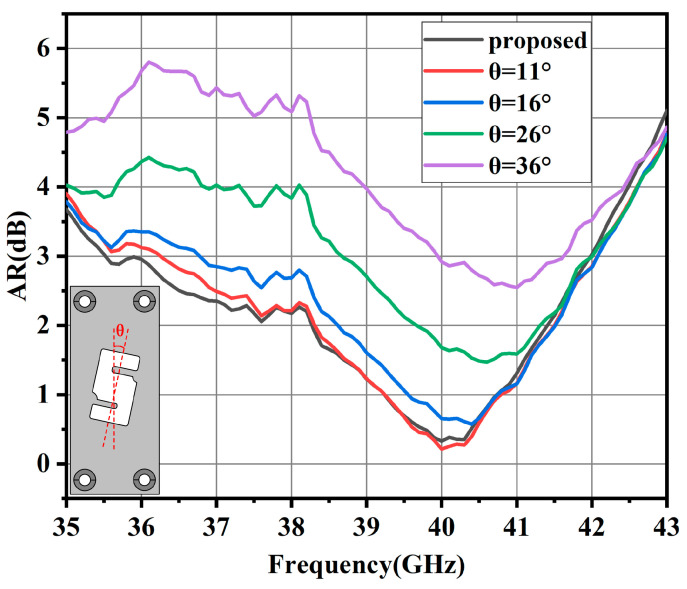
Simulated AR results with different values of θ.

In another aspect, Figure 3 illustrates the simulated axial ratio results with different placements. Note that the θ is the angle between the aluminum cavity and the y axis, which can be seen in Figure 3. The variation in the simulated AR results further indicates that the angle mentioned above is responsible for the circularly polarized performance. Finally, the best AR performance can be obtained when θ = 12°. Note that the mismatch between the metallic *S*-shaped cavity and the radiation slot allows for the E-field distribution along the y axis within the radiation slot to rotate, hence the parameter θ can be used to optimize the AR bandwidth of the slot antenna element regardless of the input impedance bandwidth.

**Figure 4:**
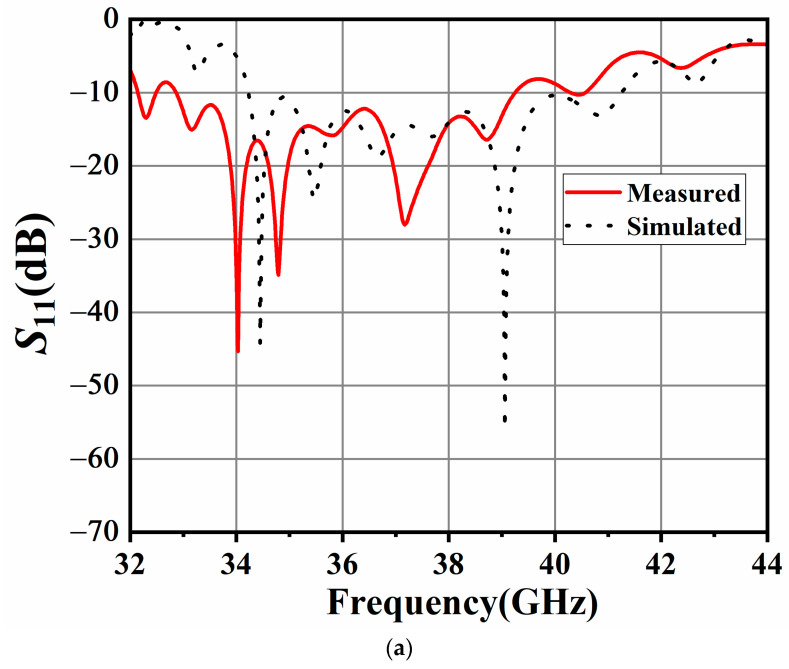

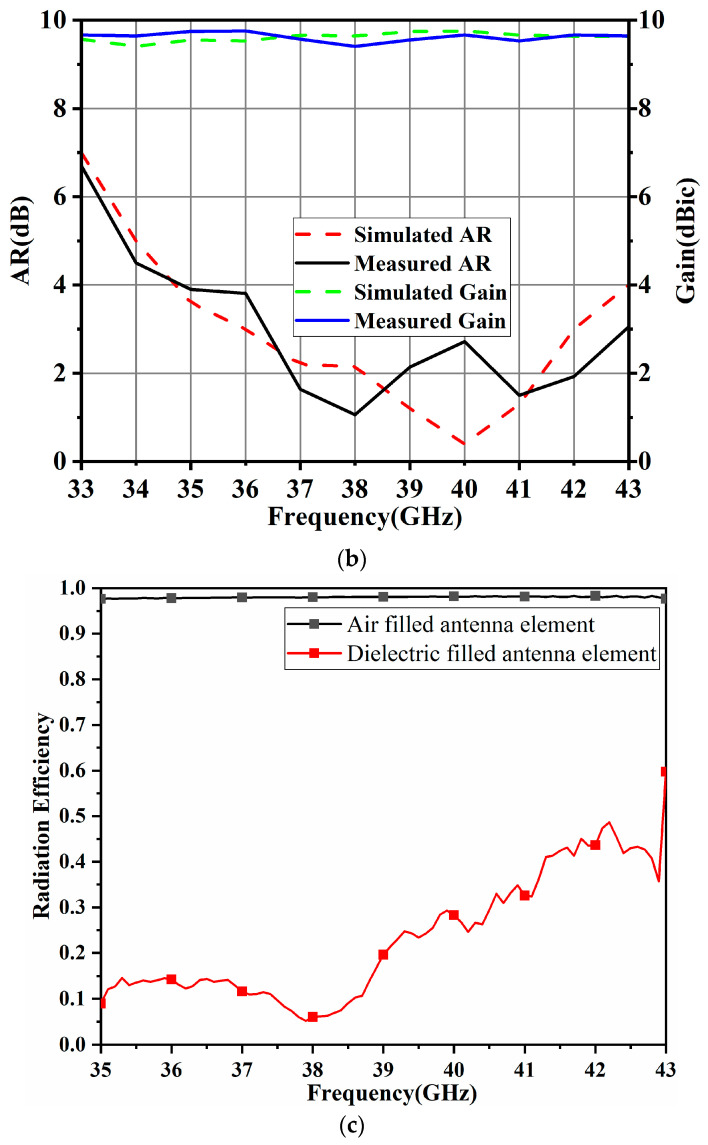
Simulated and measured results of the proposed CP antenna unit: (**a**) |S11|; (**b**) Gain and AR; (**c**) Radiation efficiency.

**Figure 5:**
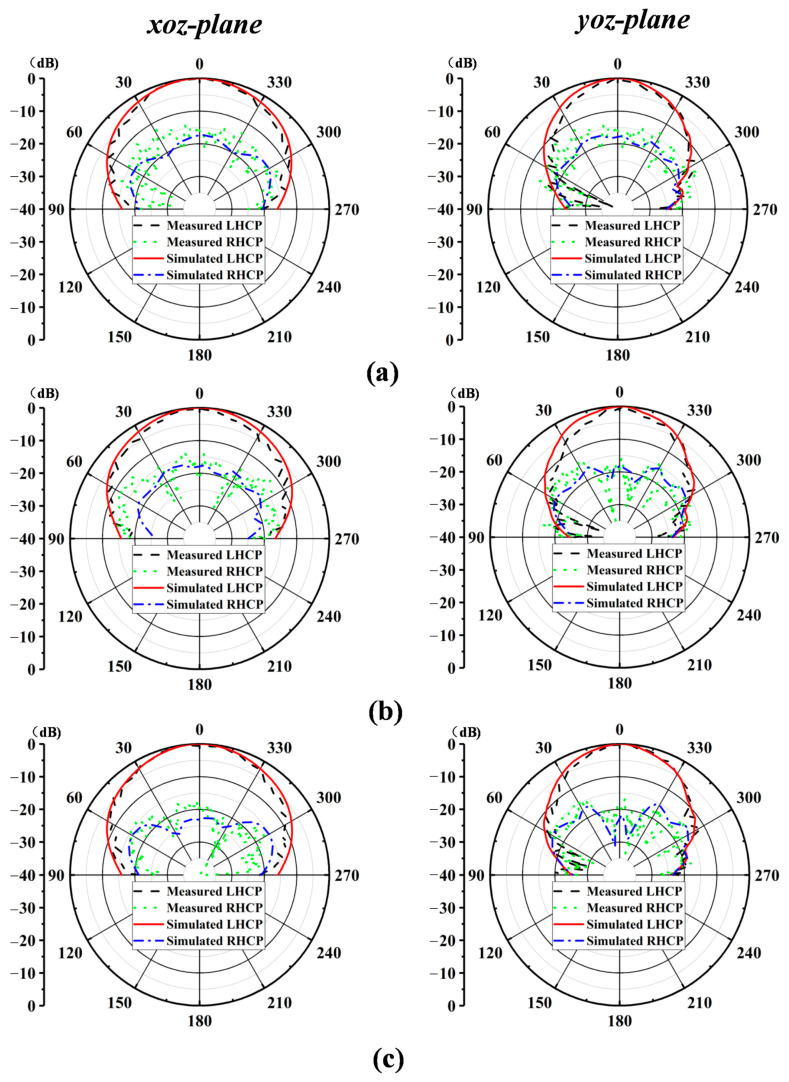
Simulated and measured radiation patterns of the proposed antenna at: (**a**) f = 37 GHz; (**b**) f = 38 GHz; (**c**) f = 39 GHz.

The proposed antenna element was fabricated, and the prototype was measured in the antenna testing chamber designed by Fragrant Mountain Microwave. Figure 4 shows the measured S_11_, AR, gain, and radiation efficiency for the proposed antenna element. The measured input impedance bandwidth is 17.7% for S11 ≤ −10 dB, ranging from 32.8 GHz to 39.2 GHz, which is slightly different from the simulated result (34.2–41.5 GHz). As for the circular polarization characteristics, the measured 3 dB AR bandwidth is 16.4% (36.5–43 GHz), and the simulated 3 dB AR bandwidth is 15.4%, ranging from 36 GHz to 42 GHz. There is a certain deviation between the simulated results and measured results, which may be attributed to the fabrication and measurement errors. Also, the realized gain of the proposed antenna element is higher than 9.5 dBic within the operating band, which could be observed in Figure 4b. Note that the realized gain within the operating band is stable, the characteristics of which are very suitable for antenna array design. As shown in the Figure 4c, compared with the dielectric (FR4, loss tangent is 0.02)-filled SIW structure, the AF-SIW (air-filled) structure proposed can reduce the dielectric loss of the antenna thus improving the efficiency. Finally, the measured and simulated results of the radiation patterns in the *xoz* and *yoz* planes at 37 GHz, 38 GHz, and 39 GHz are illustrated in Figure 5a–c, respectively. As shown in Figure 5, the antenna yields a stable left-hand CP radiation pattern in both the *xoz* and *yoz* planes at all frequencies, and the cross-polarization is smaller than −10 dB.

## 3. 1 × 8 Antenna Array Design

Considering that the path loss for the MMW band is much larger than that of a low-frequency band, the antenna array with a desirable realized gain is preferred for MMW communication applications. Hence, the 1 × 8 slot antenna array has also been designed and is studied in this section.

**Figure 6:**
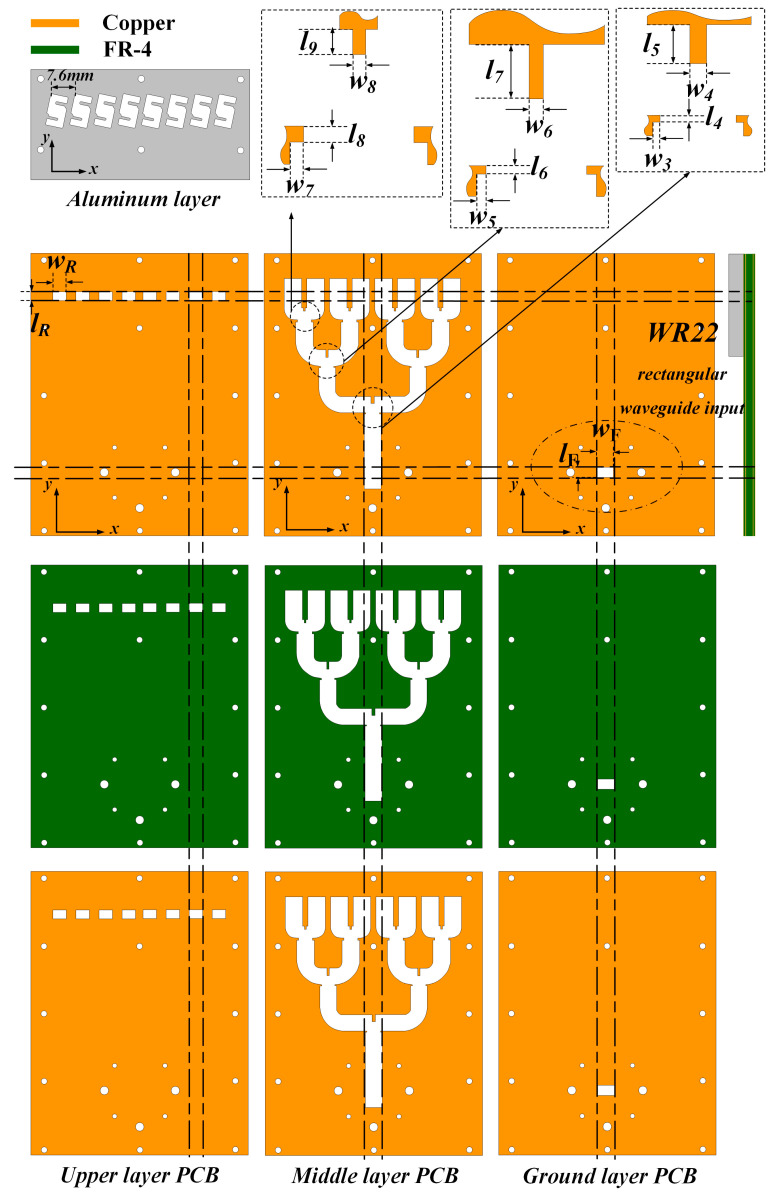
Geometry of the proposed antenna array.

**Figure 7:**
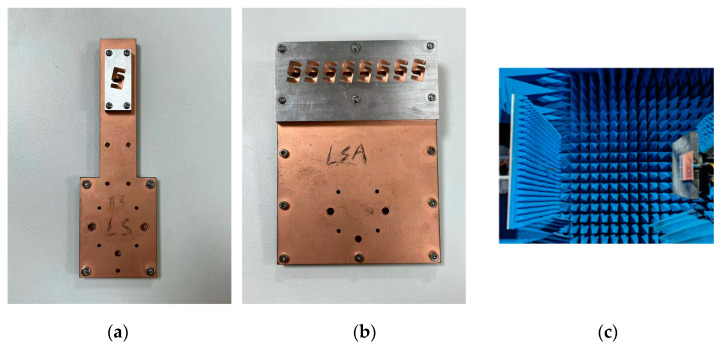
Pictures of the proposed antenna element, array, and the chamber: (**a**) CP antenna element; (**b**) CP antenna array; (**c**) microwave chamber.

Firstly, the detailed configuration as well as the key parameters of the proposed slot antenna array are depicted in Figure 6. Note that three-layered substrates are utilized to achieve the high-gain 1 × 8 slot antenna array. The proposed design is manufactured by the printed circuit board (PCB) technique, which is a low-cost solution in a higher band. As for the metallic cavity, the computerized numerical control (CNC) technique has been utilized to obtain the radiation configuration. The PCB substrate layers and the aluminum (metallic cavity) layer are fixed by screws. The overall dimension of the 1 × 8 slot antenna array including the feeding network is 96.7 mm× 70 mm × 8.41 mm. Note that the distance between the adjacent metallic cavity is 7.6 mm. Also, the 1-to-8 power divider is carefully optimized, and the parameters of *w*_3_ to *w*_8_ and *l*_4_ to *l*_9_ are listed in Table 2. The input impedance matching performance of the arrays was measured using the Keysight vector network analyzer (VNA). The radiation patterns as well as the realized gain were also measured in the anechoic chamber. Figure 7 shows the antenna configurations as well as the measured process.

**Figure 8:**
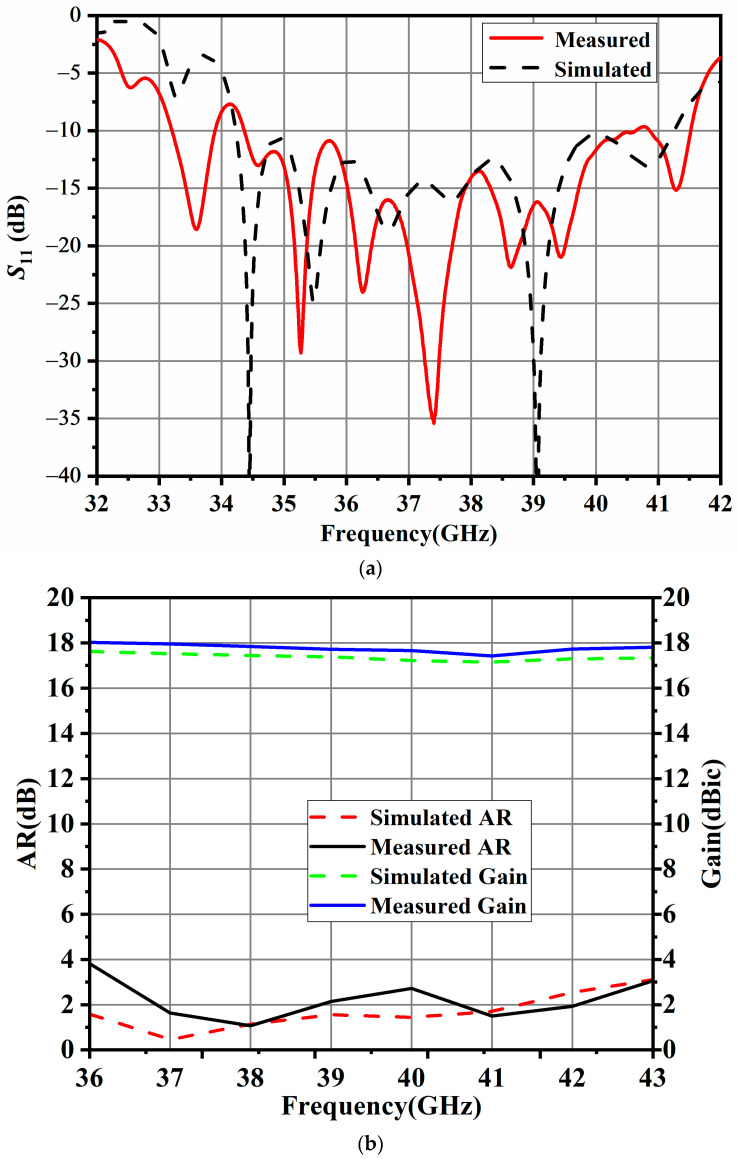
Simulated and measured results of the proposed 1 × 8 CP array antenna: (**a**) |S11|; (**b**) Gain and AR.

Simulated and measured results of the proposed antenna array are shown in Figure 8. The simulated input impedance bandwidth is 18.8% (34.2–41.3 GHz), and the measured one is 18.4% from 34.5 to 41.5 GHz, which can be observed in Figure 8a. The simulated 3 dB AR bandwidth is 17.7% (36–43 GHz), and the measured one is 16.4% (36.5–43 GHz). The measured peak gain of the proposed slot antenna array is 18 dBic at 36.5 GHz, and the 3 dB gain bandwidth is from 36 to 43 GHz in Figure 8b. The overall overlapped bandwidth is 12.8% (36.5–41.5 GHz).

**Figure 9:**
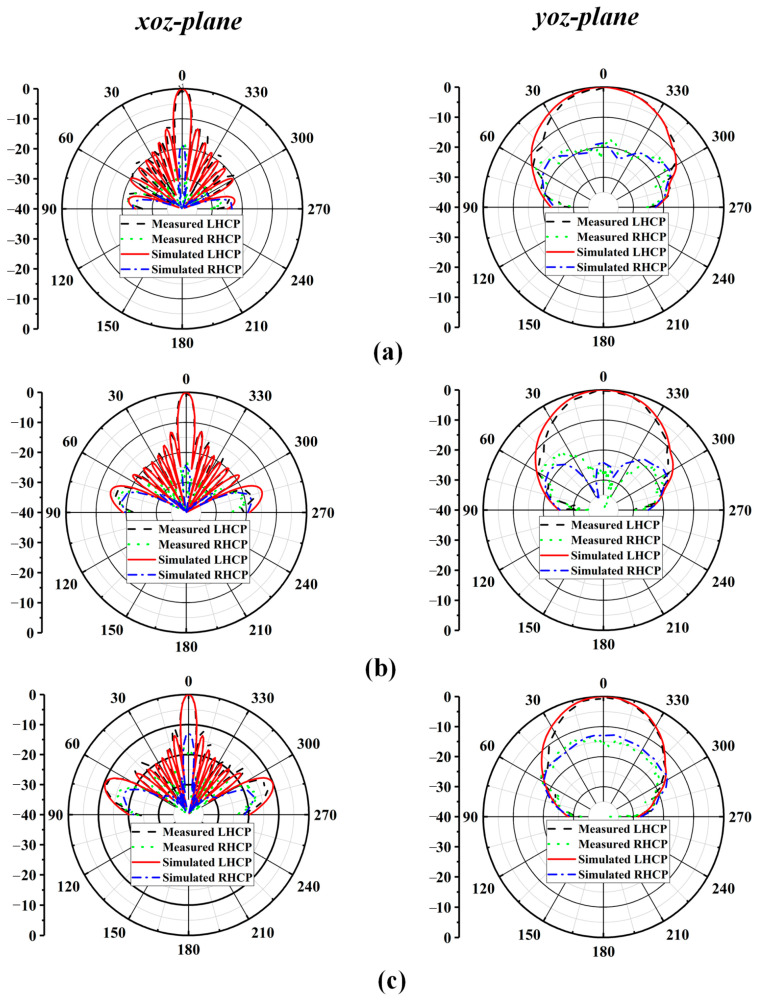
Simulated and measured radiation patterns of the proposed antenna array at: (**a**) f = 37 GHz; (**b**) f = 38 GHz; (**c**) f = 39 GHz.

Figure 9 shows the comparisons between the simulated and measured co- and cross-polarization radiation patterns for the *xoz* and *yoz* planes at 37, 38, and 39 GHz. The proposed antenna was measured using a far-field measurement system. A stable radiation pattern can be observed within the operating band. In the *xoz* and *yoz* planes, the measured radiation patterns are a good match with the simulated radiation patterns. Due to stable radiation characteristics, it can be observed from Figure 9 that the radiation patterns are generally symmetrical. Cross-polarization reaches its lowest point at the boresight direction, which is largely expected for wireless communication applications.

Table 3 presents a comparison of the proposed work as well as the reported antenna arrays. Firstly, the loss of the antenna feeding network cannot be ignored in the design of large antenna arrays. The feeding network structures with a delay line and SIW are widely used in antenna design in [7,10,12]. When comparing with these structures, it can be observed that the AF-SIW structure effectively reduces dielectric loss. Additionally, compared with the chip antenna, the metal cavity acts as the radiation structure which can avoid dielectric loss and improve the gain performance. The antenna proposed in this paper utilizes the AF-SIW as the feed structure and an all-metal structure as the radiation cavity. On the other hand, metal arms are added in metal slots to perturb the electric field and achieve circular polarization.

## 4. Conclusions

In conclusion, to address the attenuation and loss in millimeter-wave communication, this paper proposes a high-gain CP slot antenna array. The antenna achieves an impedance bandwidth of more than 18.4% and an axial ratio bandwidth of 16.4%, while achieving a high gain of 18 dBic by using AF-SIW and metal cavities. The proposed slot antenna array can cover the 39 GHz band (37–40 GHz), and the proposed array possesses several advantages such as a wideband performance, high gain, desirable radiation properties and low cost, making it an excellent choice for wideband MMW applications.

## Figures and Tables

**Table 1:** Dimensions of the antenna element.

Parameter	Value (mm)	Parameter	Value (mm)
*w*_1_	5	*l*_2_	4
*w*_2_	5.93	*l*_3_	77.49
*w*_F_	5	*l*_R_	2.5
*w*_R_	3.8	*l*_F_	2.6
*l*_1_	2		

**Table 2:** Dimensions of divider feeding network.

Parameter	Value (mm)	Parameter	Value (mm)
*w*_3_	0.67	*l*_4_	1.15
*w*_4_	6.8	*l*_5_	2
*w*_5_	3.8	*l*_6_	4.3
*w*_6_	0.5	*l*_7_	0.7
*w*_7_	4.5	*l*_8_	1.35
*w*_8_	4	*l*_9_	1

**Table 3:** Comparison of CP millimeter-wave antenna array.

Ref.	Numbers of Element	Imp.BW (%)	3-dB AR BW	Peak Gain (dBic)
[7]	4 × 4	13.7	14	18.2
[10]	4 × 4	7.3	2.8	18.5
[12]	4 × 4	8.9	8.9	19.2
[18]	2 × 2	14.75	5.4	13.59
[19]	2 × 2	23.3	7.7	10.8
[20]	2 × 2	16.3	11.9	16
This work	1 × 8	18.4	15.6	19.8

## Data Availability

Dataset available on request from the authors.
